# A simple, step-by-step dissection protocol for the rapid isolation of mouse dorsal root ganglia

**DOI:** 10.1186/s13104-016-1915-8

**Published:** 2016-02-11

**Authors:** James N. Sleigh, Greg A. Weir, Giampietro Schiavo

**Affiliations:** Sobell Department of Motor Neuroscience and Movement Disorders, Institute of Neurology, University College London, London, WC1 N 3BG UK; Nuffield Department of Clinical Neurosciences, University of Oxford, John Radcliffe Hospital, Oxford, OX3 9DU UK

**Keywords:** Dorsal root ganglia (DRG), Sensory neuron, Peripheral neuropathy, Primary culture

## Abstract

**Background:**

The cell bodies of sensory neurons, which transmit information from the external environment to the spinal cord, can be found at all levels of the spinal column in paired structures called dorsal root ganglia (DRG). Rodent DRG neurons have long been studied in the laboratory to improve understanding of sensory nerve development and function, and have been instrumental in determining mechanisms underlying pain and neurodegeneration in disorders of the peripheral nervous system. Here, we describe a simple, step-by-step protocol for the swift isolation of mouse DRG, which can be enzymatically dissociated to produce fully differentiated primary neuronal cultures, or processed for downstream analyses, such as immunohistochemistry or RNA profiling.

**Findings:**

After dissecting out the spinal column, from the base of the skull to the level of the femurs, it can be cut down the mid-line and the spinal cord and meninges removed, before extracting the DRG and detaching unwanted axons. This protocol allows the easy and rapid isolation of DRG with minimal practice and dissection experience. The process is both faster and less technically challenging than extracting the ganglia from the in situ column after performing a dorsal laminectomy.

**Conclusions:**

This approach reduces the time required to collect DRG, thereby improving efficiency, permitting less opportunity for tissue deterioration, and, ultimately, increasing the chances of generating healthy primary DRG cultures or high quality, reproducible experiments using DRG tissue.

## Background

**Fig. 1:**
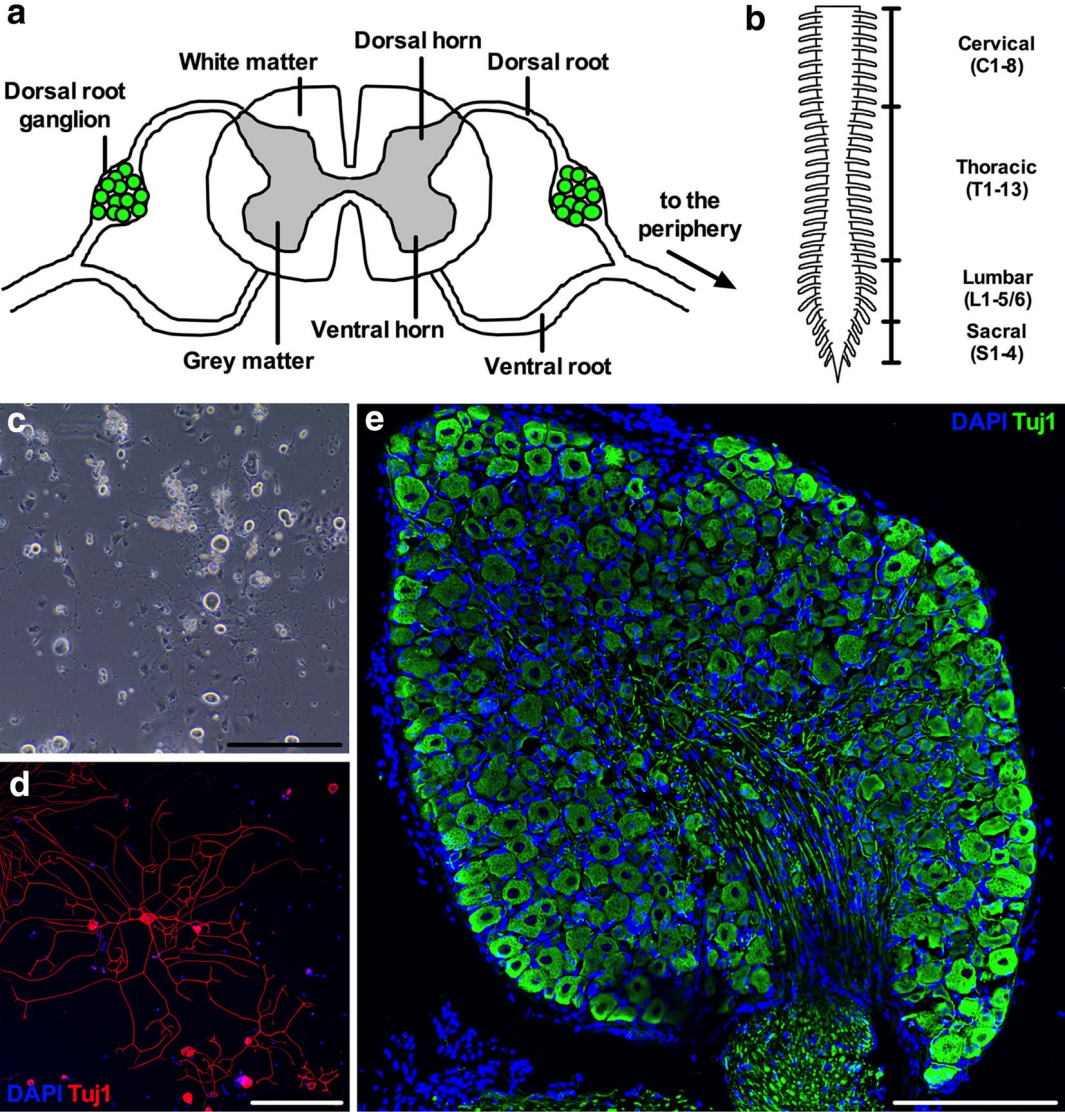
Dissected DRG can be enzymatically dissociated and grown in culture or sectioned for immunohistochemical analyses. **a** DRG are clusters of sensory neuron cell bodies located in the dorsal roots of the spinal column. The schematic portrays a transverse section of the spinal column. **b** Mice possess 8 cervical, 13 thoracic, 5 or 6 lumbar, and 4 sacral DRG pairs totalling 60 or 62 individual ganglia depending on genetic background. The picture depicts the dorsal aspect of the spinal cord and the spinal nerve roots without bilateral DRG for simplicity. **c, d** Representative images of primary DRG sensory neuron cultures 24 h post-plating. Live cultures imaged by phase contrast microscopy **c** or fixed neurons imaged by confocal microscopy **d** can be used to assess various cellular phenotypes including morphology (e.g. cell soma area and axon length), electrophysiology, and protein localisation. Note how quickly DRG neurons extend axonal processes. **e** Intact DRG can be fixed, embedded in freezing medium, sectioned on a cryostat (thickness of this example 10 μm), and analysed immunohistochemically. *β*III-tubulin (a.k.a. Tuj1) is a pan-neuronal marker, while DAPI identifies nuclei. Note that not all DAPI^+^ cells are Tuj1^+^, indicating that non-neuronal cells are present in vitro and in vivo. DRG were dissected from one month old wild-type animals (**c**–**e**). *Scale bars* 200 μm

Located in the dorsal intervertebral foramen adjacent to the spinal cord, dorsal root ganglia (DRG) are heterogeneous collections of sensory neuron cell somas found in pairs at each level of the spinal column (Fig. 1a). DRG sensory neurons are pseudo-unipolar, because they have a single axon projecting from the cell body that then bifurcates into two branches that centrally and distally target the dorsal horn of the spinal cord and peripheral tissues, respectively. Broad functional classes of sensory neurons connect with distinct regions of the spinal cord, for example pain-sensing nociceptors form synapses predominantly in superficial grey matter laminae (I–II), while touch-sensitive mechanoreceptive neurons typically terminate in deeper laminae (III–V) [1]. In order to perceive diverse stimuli from the environment, functional sensory subtypes target different regions of the periphery, for instance proprioceptive neurons, which are important for sensing body position in space, innervate muscle spindles, while temperature-sensing thermoceptive cells often end in the skin [2]. The distal ends of sensory nerves are consequently specialised, culminating in or synapsing with assorted sense organs adapted to function, for example Pacinian corpuscles for pressure perception or Golgi tendon organs for detecting muscle tension [3]. The diverse repertoire of DRG sensory neuron varieties has been classified based on how cellular characteristics such as electrophysiological properties, growth factor response, morphology, and protein markers relate to function [4–7]. However, recent single-cell, RNA sequencing experiments indicate that there are potentially 11 distinct afferent subtypes based on discrete molecular identities and function [8].

Rodent DRG neurons have been used to study sensory nerve development, function, and regeneration [9–11], to aid in drug discovery [12], and to elucidate the mechanisms underlying peripheral nerve disorders, such as diabetic neuropathy [13, 14] and Charcot-Marie-Tooth disease [15, 16]. Dependent on genetic background, mice possess 30 or 31 pairs of DRG—8 cervical, 13 thoracic, 5 or 6 lumbar and 4 sacral (Fig. 1b) [17–19]. By axotomy of associated axon bundles, DRG can be carefully removed from healthy or disease model mice and then be enzymatically disrupted to produce live sensory neuron cultures (Fig. 1c, d), or fixed, sectioned, and immunohistochemically analysed (Fig. 1e). Importantly, the cultured sensory neurons faithfully retain features of the in vivo neurons and reflect the diverse population of DRG cells at the time of dissection [20]. DRG can alternatively be processed for other downstream experiments, such as protein or RNA analyses [21, 22].

Here, we describe a simple and rapid technique for dissecting DRG from the spinal column of postnatal day 5 (P5) mice or older. This protocol can be combined with previously published culturing methods [17, 23–25], immunohistochemistry techniques [26, 27], or RNA (e.g. reverse transcriptase PCR) and protein (e.g. western blotting) evaluation [28], for in depth sensory nerve analyses.

## Methods

### General

**Fig. 2:**
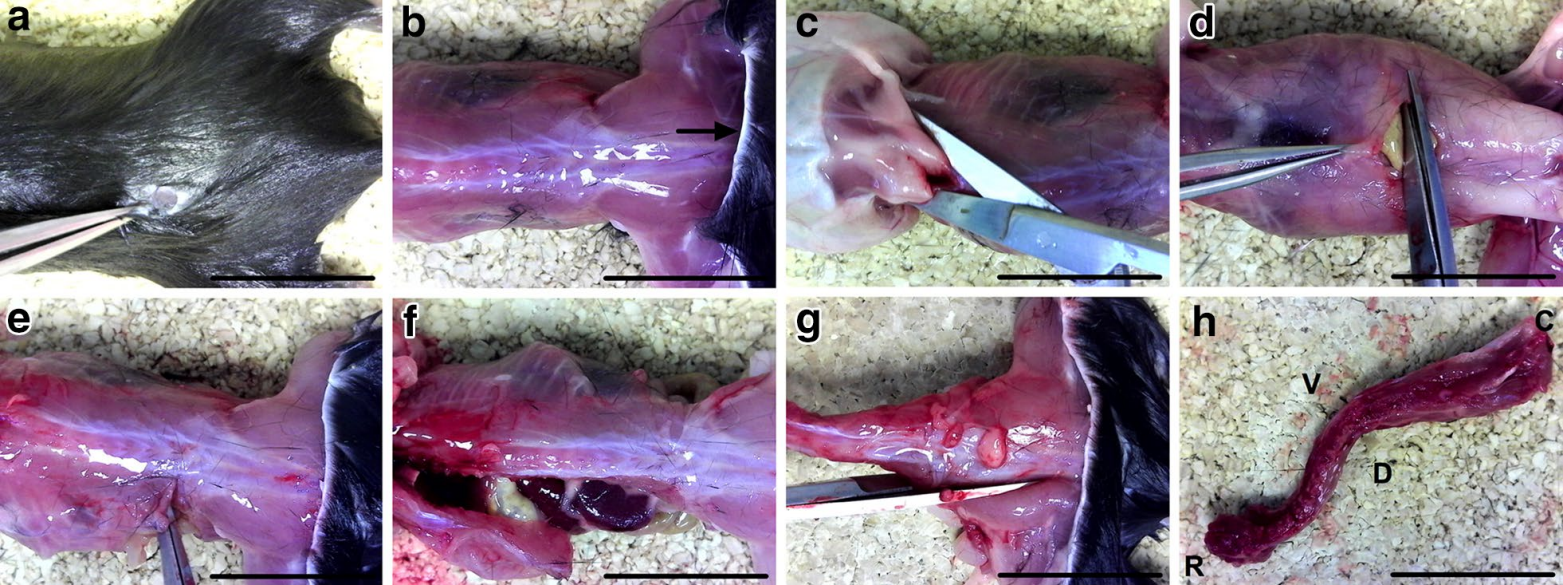
Spinal column isolation. **a, b** After dousing the fur with 70 % ethanol, a small incision is made in the dorsal skin at the level of the hips (**a**), and the pelt (*arrow*) removed from the head to hind limbs (**b**). **c** The head is removed by cutting at the base of the skull (C1–2 level) and the arms are cut beneath the shoulders to aid removal of the skin. **d, e** An incision is made through the abdominal wall muscles (**d**) and continued laterally to the spinal column in both directions (**e**). **f** The ribs are then cut parallel with and close to the spinal column on both sides, before detaching the viscera connected to the anterior side of the spinal column. **g, h** The femurs are cut (**g**), and the spinal column removed (**h**) by making a transverse cut at the level of the femurs. In all panels, the head is to the left and tail to the right. The dorsal aspect is imaged in all panels except for **d** (ventral aspect) and **h** (*C* caudal, *D* dorsal, *R* rostral, *V* ventral). *Scale bars* 2 cm

**Fig. 3:**
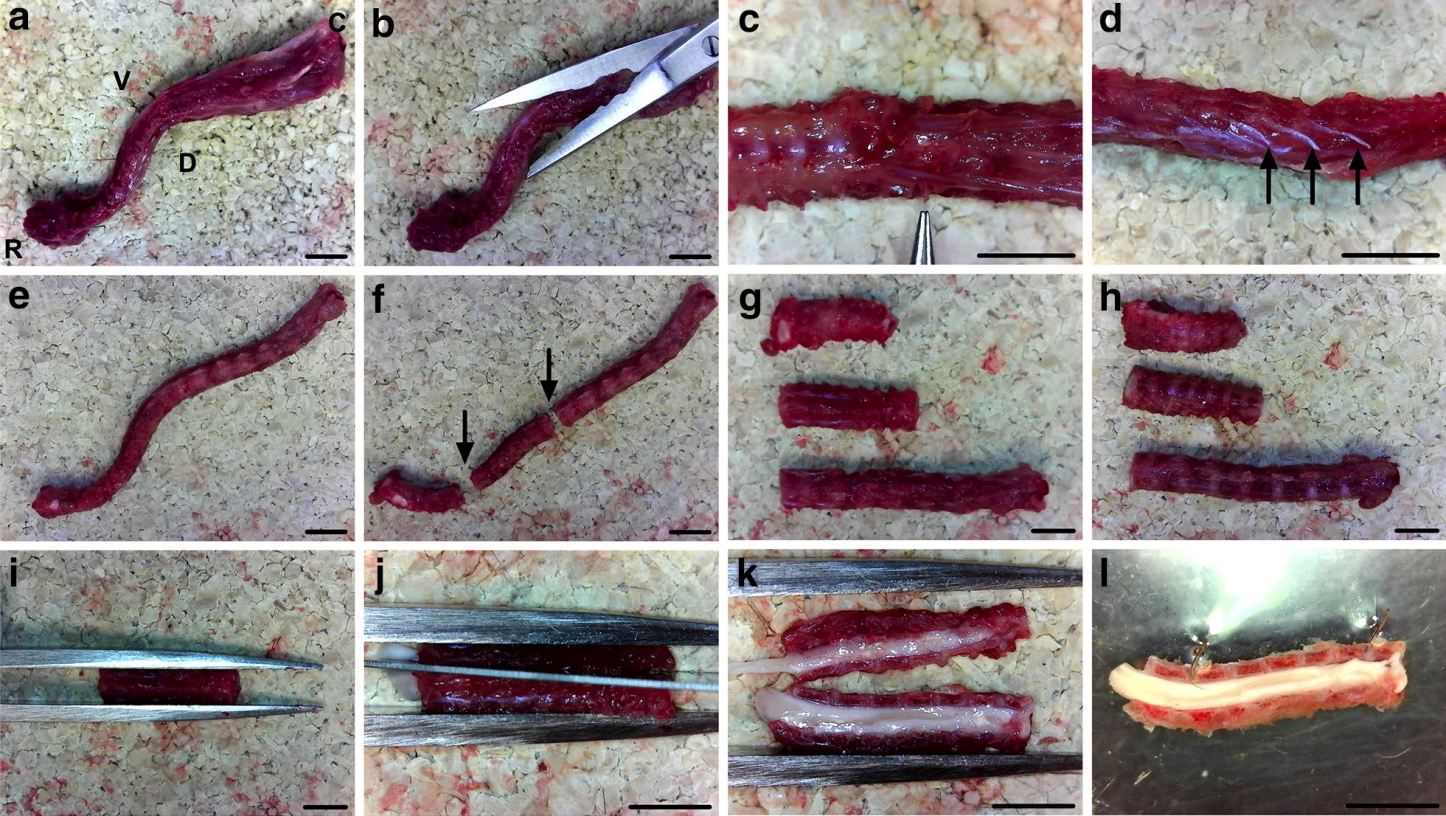
Spinal cord exposure. **a, b** Muscle, fat, and other soft tissues are cut from the spinal column using curved scissors. **c** The T13 level DRG pair is found caudal to the most caudal ribs, which are used as landmarks (identified by forceps). **d** Spinal nerves (*arrows*) project from the column and can be removed. **e, f** Once cleared of soft tissues (**e**), the column can be cut (*arrows*) into three pieces (**f**), with one cut at the level of the last rib in order to orientate the dissection. **g, h** The column segments must be placed dorsal (**g**), not ventral (**h**), side facing up. **i, j, k** Thick forceps are then used to secure the spinal column dorsal side up (**i**), before cutting it into two equal halves along the midline (**j, k**). **l** The spinal column hemi-segments can be pinned out, medial side up, using two insect pins through intervertebral discs in Sylguard-lined petri dishes, before flooding with ice cold PBS. In all *panels*, the rostral end is to the left and the caudal end to the right. *Scale bars* 0.5 cm

**Fig. 4:**
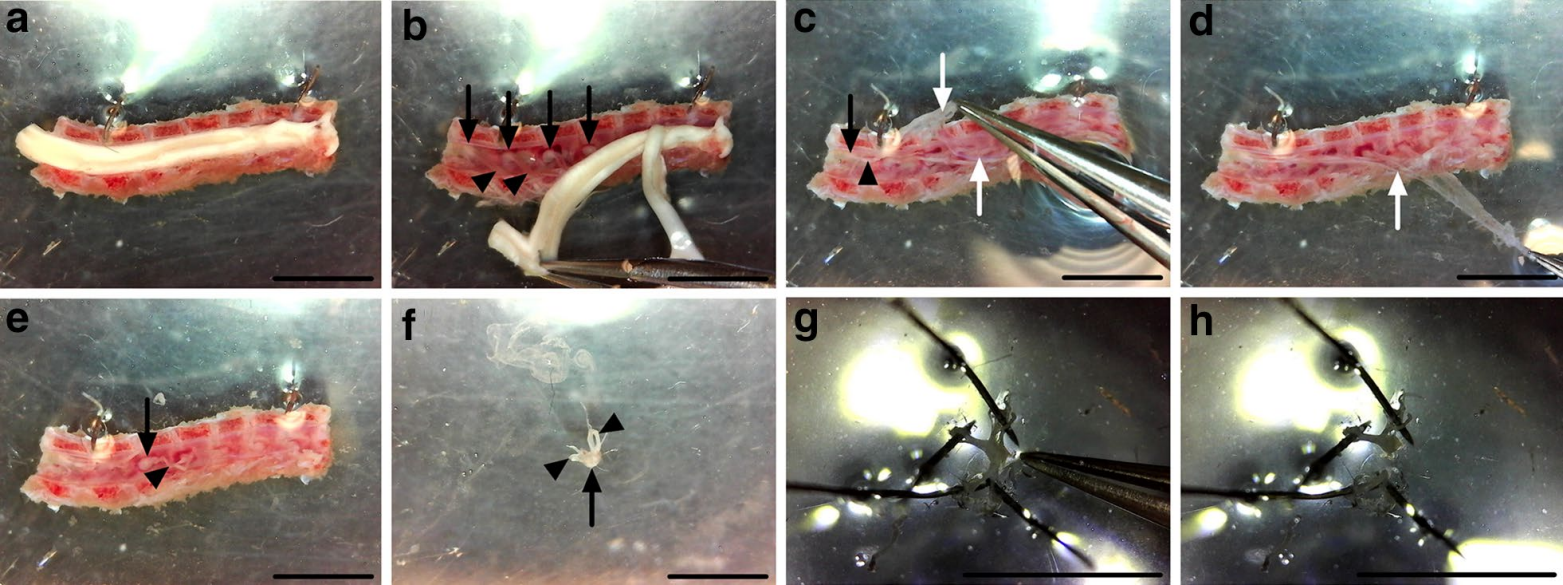
DRG extraction and cleaning. **a, b** The spinal cord is peeled from the pinned column in a rostral to caudal direction. **c–e** The meninges are identified as translucent sheets of tissue covering the DRG (**c**), and carefully removed (**d**), making the DRG easier to see (**e**). **f** Individual ganglia are extricated by clasping and lifting with forceps the distally projecting axon bundles found on the lateral side of the DRG. Care must be taken not to damage the DRG with the forceps. **g, h** DRG are then pinned out via their axons, and any residual meninges removed (**g**), before cutting the axons close to the DRG (**h**). In all *panels*, the rostral end is to the left and the caudal end to the right, and *black arrows* and *arrowheads* highlight DRG and axon bundles, respectively, while *white arrows* identify the meninges. In **a–e**, the medial aspect is facing up. *Scale bars* 0.5 cm

The mouse imaged in Figs. 2, 3, 4 was a 3 month old (P86) wild-type C57BL/6 male. Mouse sensory neurons can be dissected and cultured as soon as they are formed in the embryo [about embryonic day 13 (E13)] [29]; however, this particular dissection protocol can only be used on mice approximately P5 and above (the older and larger the animal, the easier the dissection). Protocols to dissect embryonic rodent DRG are available [30, 31]. Images of in vitro and ex vivo DRG neurons were taken using an EVOS™ XL Core Cell Imaging System (Thermo Fisher, Fig. 1c) and a Zeiss LSM 780 laser scanning microscope (Fig. 1d, e), respectively, while dissection images (Figs. 2, 3, 4) were taken with an eFlex™ digital microscope (Carson Optical, MM-840). Scale bars in images taken using the EVOS™ system were incorporated by imaging a ruler in parallel images, and are thus approximate. To decrease blood contamination, animals can be transcardially perfused with saline or phosphate buffered saline (PBS) prior to dissection. This is not necessary for generating healthy, contamination-free primary cultures or immunohistochemical tissue sections of high clarity; however, perfusion is likely to improve specificity of proteomic or RNA analyses. Dissections to produce primary cultures should be performed in a laminar flow hood under sterile conditions to reduce the chances of contamination; nevertheless, in our experience, dissection under the hood is not strictly required. To preserve neuronal health, DRG should be kept as cold as possible throughout the dissection. A modified version of this protocol is available for the isolation of adult rat DRG [32].

### Reagents, equipment and set up

The following materials and reagents (Sigma, unless otherwise stated), or similar alternatives, are required for the dissection: bone scissors (Fine Science Tools [FST], 14110-15), fine curved and straight scissors (FST, 14095-11 and 14094-11), small spring scissors (FST, 15000-08), thick and fine forceps (FST, 11000-25 and 11251-10), 70 % (v/v) ethanol in distilled water, fine marker pen, 12 × 0.2 mm minutiens insect pins (Austerlitz, 0.20), PBS (137 mM NaCl, 10 mM Na_2_HPO_4_, 2.7 mM KCl, 2 mM KH_2_PO_4_), Hank’s balanced salt solution (HBSS, Thermo Fisher, 14170-112), 60 × 15 mm petri dishes (BD Biosciences, 351007) lined with Sylguard 184 silicone elastomer (Dow Corning, 01015311), disposable surgical scalpel blade (Swann-Morton, 0208), and SZB 250 dissection microscope (VWR, 630-1577). Sylguard 184 silicone elastomer was prepared by combining the elastomer base with the curing agent (10:1), using the mix to line petri dishes, and allowing to set for at least 48 h. To remove bubbles from the Sylguard, a vacuum desiccator can be used before setting.

### Dissection protocol

#### Animals

All animal handling and experiments conformed to the Home Office Animals (Scientific Procedures) Act (1986) and were approved by the University College London—Institute of Neurology Ethics Committee. Animals were sacrificed using carbon dioxide before confirmation of death, rather than cervical dislocation, as the latter may damage cervical DRG.

#### Isolation of the spinal column

To restrict fur contamination, the deceased animal should be doused with 70 % ethanol. The torso may also be shaved (prior to this); however, this is not necessary. Forceps are used to pinch the external layer of fur and skin, while a small, dorsal incision is made using fine scissors in the region of the pelvis (Fig. 2a). The pelt is then removed from the head to the base of the tail (Fig. 2b), by either cutting or careful tearing of the skin in the transverse plane, followed by pulling the pelt up and over the head. The arms and head are then removed by cutting with scissors beneath the shoulder blades and at the C1–2 region of the column, found adjacent to the base of the skull (Fig. 2c). The abdominal wall musculature is cut on the ventral side (Fig. 2d) and continued laterally, one direction at a time, until the spinal column is reached (Fig. 2e). The scissors are then turned at right angles to face the rostral direction, and all ribs are detached close to the spinal column on both sides (Fig. 2f). At this point, a fine marker pen can be used to highlight the most caudal ribs (Fig. 3c), which act as a landmark for the T13 ganglia found just caudal to the ribs [17]. The diaphragm, viscera, and rib cage are removed from the anterior side of the column. The femurs are then cut using bone scissors close to the column (Fig. 2g), and the whole spinal column removed (Fig. 2h) by making a transverse cut at the level of the femurs.

#### Exposure of the spinal cord

Once the spinal column has been excised (Fig. 3a), extraneous muscle, fat, spinal nerves and other soft tissue found on the exterior of the column are removed using fine curved scissors with the blade tips facing up (Fig. 3b–e), reducing the likelihood of accidentally cutting into the column. The column is then cut in the transverse plane into three pieces, with one cut just below the most caudal rib to orientate the spinal cord region (Fig. 3f). To limit the chances of damaging a DRG pair, try to perform transverse cuts through the vertebrae between the discs. These preceding steps (Fig. 3a–f) are performed to facilitate the next step of halving the spinal column. Thick forceps are used to hold the spinal column segments straight and steady, dorsal side facing up, while a sterile surgical scalpel blade is used to divide the spinal column in half down the midline (Fig. 3g–k). It is vital that this cut is done as close to the midline as possible, in order to improve subsequent access to the DRG. A rolling motion along the midline, starting at one end of the segment and finishing at the other, using a curved scalpel blade is perhaps the easiest way to assure accurate cutting. The more soft tissue removed from the exterior of the column, the easier this cutting process will be. Using a dissection scope, the two halves of the spinal column are then pinned out in Sylguard-lined petri dishes, medial side facing up, using insect pins through the exterior intervertebral discs. Sterile, ice cold PBS is then added to the petri dish to aid dissection and keep the sample from desiccation. To keep the dissection cold, PBS should be regularly replaced.

#### Extraction and cleaning of DRG

The spinal cord can now be slowly peeled in a rostral to caudal direction from the column, revealing the DRG below (Fig. 4a, b). Care should be taken at this point not to remove all ganglia with the spinal cord, as this will complicate DRG identification, dissection, and cleaning. To prevent this, the underlying meninges are held with fine forceps if they begin to be pulled away with the cord. Once the cord has been discarded, the meninges must be identified and detached in order to reduce cellular contamination of cultures and restrict sample rolling when sectioning on the cryostat (Fig. 4c–e). The meninges surround the spinal cord in situ, and cover DRG once the cord is taken out. To extract the meninges, use fine forceps to carefully peel them back from one end of the spinal column segment to the other. If they prove difficult to identify, try grasping at the vertebrae between the DRG using fine forceps. Sometimes the DRG are extracted with the meninges. This is not a problem, just pin out the meninges using insect pins and carefully extricate the individual DRG. If the DRG remain in the column, use fine forceps to grasp the distally projecting axon bundle found on the lateral side of the ganglia, and lift the DRG up and out of the column taking care not to touch and damage the cell ganglion at all times. DRG can then be pinned out using their axons in the Sylguard (Fig. 4f), before removing residual meninges (Fig. 4g), and using fine spring scissors to cut away the axon bundles found on the outside of the DRG (Fig. 4h). This last step will reduce myelin debris and glial cell contamination, which is important for both culturing and protein/RNA profiling. The long and thin white axons are easily distinguished from the round and darker DRG. Once dissected and cleaned, DRG can then be collected in HBSS for subsequent enzymatic digestion of the extracellular matrix and culturing (Fig. 1c, d), fixed for sectioning and immunohistochemistry (Fig. 1e), or processed for assessment of protein/RNA levels [21, 22].

## Discussion

Dissection of up to 40 individual DRG, from the time of animal euthanisation to commencing enzyme treatment or fixation, can be easily completed in 20–30 min per animal using this simple dissection protocol. The method is technically easier to perfect and, in our hands, considerably quicker than performing a spinal cord laminectomy with subsequent DRG removal from the in situ spinal column, which appears to be the most common dissection method described in the literature [17, 24, 32, 33]. A limitation specific to the dissection technique outlined here is that is requires the cutting of the spinal cord down the midline, which may affect its subsequent analysis if required. Nonetheless, the reduced dissection time diminishes the opportunity for cellular damage of DRG neurons, leads to a greater preservation of neuronal viability, and therefore increases the possibility of performing accurate and reproducible downstream experiments. Given its simplicity, the technique can be quickly perfected with little practice and anatomical knowledge by researchers of any dissection experience.

Examination of DRG neurons has provided a wealth of information on the development, regeneration, and function of not only sensory nerves, but the nervous system as a whole. Moreover, in combination with analyses of the neuromuscular system, for example neuromuscular junction phenotyping [34, 35] and primary embryonic motor neuron cultures [36, 37], assessment of in vitro sensory neurons or ex vivo DRG can provide a powerful approach to improving understanding of the pathological cellular events underlying disorders of the peripheral nervous system.
